# Further evidence for the role of temporal contiguity as a determinant of overshadowing

**DOI:** 10.1177/17470218231197170

**Published:** 2023-09-18

**Authors:** José A Alcalá, Pedro M Ogallar, José Prados, Gonzalo P Urcelay

**Affiliations:** 1 University of Nottingham, Nottingham, UK; 2 Complutense University of Madrid, Madrid, Spain; 3 University of Jaén, Jaén, Spain; 4 University of Derby, Derby, UK

**Keywords:** Overshadowing, auditory signal, avoidance learning, configural processing, contiguity

## Abstract

Three experiments explored whether weakening temporal contiguity between auditory cues and an aversive outcome attenuated cue competition in an avoidance learning task with human participants. Overall, with strong temporal contiguity between auditory cues and the outcome during training (the offset of the predictive auditory signals concurred with the onset of the outcome), the target cue trained as part of a compound yielded less avoidance behaviour than the control cue trained alone, an instance of overshadowing. However, weakening temporal contiguity during training (inserting a 5-s trace) attenuated overshadowing, resulting in similar avoidance behaviour in response to the control and target cues. These results provide evidence that, as predicted by a recent modification of Pearce’s configural theory, temporal contiguity is critical for determining cue competition.

In the natural environment, a given event can have multiple causes and organisms use a diversity of antecedents as potential predictors for relevant outcomes. For example, to estimate the probability of raining we can use information relative to humidity, the colour of the clouds, the barometric pressure, or the odour in the air. All these cues are informative about the outcome (rain) and can be useful for deciding whether to take an umbrella or not. However, our decision making is subject to time and cognitive resources constraints, so we might not be able to actually consider all the relevant factors before making up our mind; therefore, we tend to focus on the cue or cues we consider to be the best predictor(s) at the expense of the other cues.

Unsurprisingly, in a straightforward scenario where a single cue (e.g., A) is repeatedly paired with an outcome, it acquires strong predictive value—and therefore, capacity to influence the behaviour of the individual. However, if during training, a different target cue, X, is presented alongside a competitor cue (e.g., B) paired with the same outcome as A, its predictive value is attenuated in comparison with the control cue A trained by itself (in particular if the salience or perceptual effectiveness of B is high; see Mackintosh, 1976). Lower predictive value (and behavioural control) as a result of training in compound with a second cue is known as overshadowing (e.g., Pavlov, 1927), an example of cue competition. However, under some circumstances the presence of a second cue enhances the predictive value of the target cue, resulting in facilitation or potentiation rather than competition (e.g., Alcalá, Kirkden, et al., 2023; Batsell et al., 2012; Richardson et al., 2021; Sansa et al., 2007; Urcelay & Miller, 2009). Overshadowing and potentiation, opposite phenomena that can be observed using the same experimental design, constitute a challenge for standard associative learning theories, which were developed in the light of cue competition phenomena; a paradigmatic example would be the Rescorla–Wagner model (e.g., Rescorla & Wagner, 1972). Urcelay (2017) suggested that the differential potential outcomes of compound conditioning represent the extreme points of a behavioural continuum, from competition to facilitation with no interaction between cues in the intermediate zone of the continuum (e.g., Maes et al., 2016; Packheiser et al., 2020). This applies not just to overshadowing but also to the other main cue competition effect, blocking (e.g., Kamin, 1969), where the reverse—augmentation—has also been documented (e.g., Batsell & Batson, 1999; Vadillo & Matute, 2010, 2011). The work reported here aims to further our understanding of the key variables that determine the outcome of interactions between cues (overshadowing, potentiation, or no interaction).

One of the variables that determines whether cues will compete or facilitate each other is the temporal contiguity between events (i.e., the time elapsed between cue and outcome). Across species and learning domains (see Urcelay, 2017), studies have revealed that weakening of temporal contiguity attenuates competition (Herrera et al., 2022), and sometimes results in a complete shift to facilitation (Alcalá, Kirkden, et al., 2023; Batsell et al., 2012; Cunha et al., 2015; Schachtman et al., 1987; Urcelay & Miller, 2009). For instance, Herrera and colleagues (2022) recently observed overshadowing with strong contiguity (i.e., the offset of the cue/s overlapped with the onset of the outcome) in an avoidance learning task with humans. In these experiments, using a within-subjects design, a control cue trained alone was found to have more behavioural control (predictive value) than a target cue trained in compound with a competitor. However, inserting a temporal interval between the offset of the cue and the onset of the outcome abolished competition: the control cue and the target cue yielded similar behavioural control. Herrera et al. (2022) also found a similar pattern of results in a navigation task in which the participants had to learn the location of a goal by reference to the geometry of a kite-shaped arena and some landmarks (the colour of the walls). Strong spatial contiguity between the cues (i.e., the landmark and the target corner where the goal was located) and the outcome (i.e., a hidden goal) resulted in overshadowing (the landmarks overshadowed the geometry of the arena), but weak contiguity (moving the goal away from the target corner of the arena and the distinctive landmark) yielded no interaction between landmarks and geometry. Overall, the authors concluded that contiguity (spatial and temporal) is necessary for competition to occur, because weakening of such contiguity (temporal or spatial) resulted in the absence of competition. To account for these findings, Herrera and colleagues (2022) proposed a tentative explanation to account for cue interactions as a function of contiguity. Building on Pearce’s configural theory (Pearce, 1987), they proposed that weakening contiguity may lead to increased configural processing that ultimately results in less generalisation decrement—from the training compound to the test cue—at the time of test. Although the details are beyond the scope of this introduction (see section “General discussion”), this modification enables Pearce’s configural model to account for not only the findings of Herrera et al. (2022), but also for recent results that revealed potentiation of Action–Outcome learning by intervening cues presented contingent on participants’ responses with weak temporal contiguity between Action and Outcome (Alcalá, Kirkden, et al., 2023).

The present study extends our previous work, aiming to provide further evidence of the role played by temporal contiguity in determining the outcome of interactions between cues. An explicit prediction of the proposal by Herrera and colleagues (2022) is that, at some point, competition should shift to facilitation. Despite the fact that—with weak contiguity—the absence of competition was consistent across experiments using different tasks and parametric variations, potentiation was not observed. In our previous work, perhaps the use of discrete visual stimuli prevented the configural processing that would lead to facilitation. The authors’ suggestion was that there are many variables determining how participants encode and process the information, encouraging either a *more* elemental or configural approach (see Melchers et al., 2008). In the avoidance learning experiments reported by Herrera et al. (2022, Experiments 1–4), four coloured sensors displayed at the top of a computer screen were used as predictive cues. In these experiments, a cue trained in compound could be formed by three pink sensors (B) and one white sensor (X). Importantly, the position of the target cue (i.e., the white sensor) changed across trials. Although the sensors’ position was objectively irrelevant during the task, participants might have attempted to make sense of the positional information, resulting in a noisy manipulation compared with the consistent use of only a single dimension (i.e., colour or location). Thus, the use of multiple locations might have promoted the elemental—rather than configural—processing of the cues.

In the current experiments, we sought to further assess how contiguity affects cue competition using the same task used by Herrera and colleagues (2022; Experiments 1–4) but using auditory instead of visual cues. The reasons to use auditory stimuli were twofold. First, in rodent studies, potentiation has been observed using auditory stimuli (Urcelay & Miller, 2009); it could be the case that this type of stimuli is more prone to configural processing than the visual stimuli used in previous experiments. As mentioned above, elemental processing of the predictive visual cues with changing locations might have prevented the establishment of configural representations, hindering the chances of observing facilitation effects. Using auditory stimuli, the BX compound emerges from the same spatial source (i.e., headphones), increasing the chances for the configural processing that leads to facilitation effects. Second, this manipulation would enable the generalisation of previous results to stimuli of a different modality, asserting the generality of the findings reported by Herrera et al. (2022). Although this might seem trivial, the prediction that contiguity modulates cue interaction has not received much attention in the field, so it is important to assess the extent to which similar findings can be observed across different sensory modalities. Moreover, unlike non-human studies in which overshadowing with auditory stimuli has been consistently observed (Jones & Haselgrove, 2011, 2013; Urcelay & Miller, 2009), in the case of human predictive learning most of the research on overshadowing has been conducted using visual stimuli (e.g., Price & Frank Yates, 1993; see Stahlman et al., 2018, for an example using visual and haptic dimensions). As far as we know, there are no demonstrations of overshadowing in humans using compounds of auditory stimuli.

To recap, in the current study we aimed to replicate the findings by Herrera et al. (2022), but using auditory stimuli. Based on previous results, we expected to find a reliable overshadowing effect with strong temporal contiguity (Experiments 1 and 3), and reduced overshadowing with weak temporal contiguity (Experiments 2 and 3). However, if auditory stimuli are more configurable than the visual stimuli previously used, it could facilitate the observation of potentiation under conditions of weak contiguity, as it was the case with rodents (Urcelay & Miller, 2009).

## Experiment 1

The first experiment sought to assess cue competition using auditory stimuli in human participants using the same task used by Herrera et al. (2022, Experiments 1–4). During the training phase, participants were exposed to a control cue A+ and a compound BX+ (where A, B, and X refer to different auditory cues and + refers to the outcome). These reinforced cues predicted the appearance of an aversive outcome. We used two additional cues as fillers, E− and FG−, which were never paired with the outcome (− indicates the absence of the outcome). In this experiment, the predictive cues and the outcome were presented with strong temporal contiguity: the outcome appeared immediately after the offset of the auditory signals. After training, the control cue A (trained alone) and the target cue X (trained in compound with B) were tested. We expected to observe a deficit in the behavioural control yielded by X compared with A, that is, an overshadowing effect.

### Method

#### Participants

A total of 28 undergraduate students at the University of Leicester (23 female, 5 men), with an average age of 19.82 years (*SD* = 2.60; range = 18–29) participated in the experiment in exchange for course credit. The participants had no previous experience with the task. The experimental protocol was approved by the Psychology Ethics Committee of the University of Leicester.

The sample size was based on previous work using this protocol (Herrera et al., 2022). Sensitivity analyses using the software G*power revealed that with a sample of 28 participants, the smallest effect size that could be detected for the simple effect of Signal in the last second of presentation of the predictive cues was *F*(1, 27) = 4.21, η^2^_p_ = .07, with a power of .90 and an alpha criterion of .05. This effect is smaller than the actual effect size observed, suggesting strong sensitivity in our sample to observe competition.

#### Apparatus and stimuli

Participants were tested in individual cubicles at the University of Leicester. Each cubicle contained a 19-in. AG Neovo F-419 LCD screen connected to a Hewlett-Packard Compaq Elite 8300 PC desktop computer, running Microsoft Windows 7.

**Figure 1.**
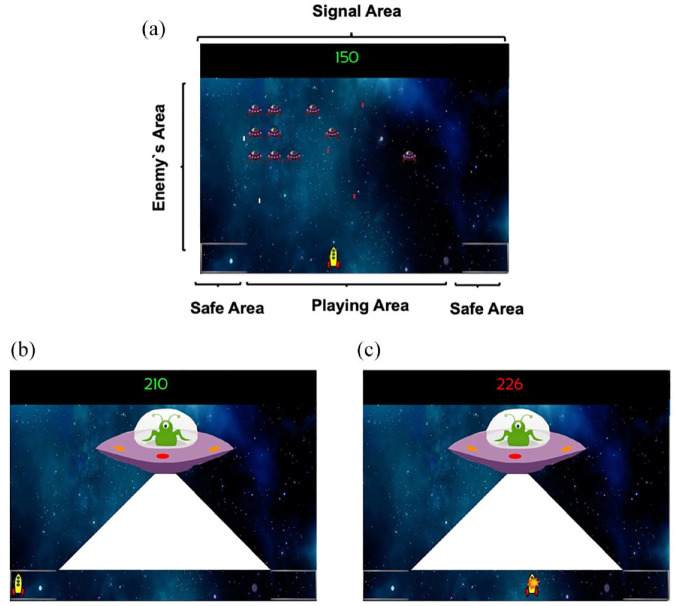
Experimental task snapshots. (a) Players’ experience during the ITI and the Trace. (b) Aversive outcome when the player is in the safe area without losing points. (c) Aversive outcome when the player is in the playing area and losing points. Dwell time in the safe area was used as a dependent measure.

The task used in this experimental series was identical to that described by Herrera et al. (2022, Experiments 1–4), except for the use of auditory rather than visual stimuli as the predictive cues. Participants had to play a version of the classic 2D game “Space Invaders” with instructions asking them to win as many points as possible during the game. The screen’s background was a picture of a fictitious galaxy and remained constant throughout the game. There were three main areas during the game: the playing area, the safety area, and the enemy’s area (see Figure 1a).

The playing area was the horizontal bottom line of the screen. The participant’s spaceship could be controlled with the left and right arrows of the keyboard and could freely move sideways along this area. By pressing the space bar, the participant’s ship shot a green laser that could destroy enemy ships, adding points to the counter on the top of the screen. The enemy ships could fire red laser shots that resulted in a deduction of points on the counter when hitting the participant’s ship. Two safe areas were located at either corner of the playing area along the bottom line, signalled by a line simulating a shield. Participants could move freely between the playing and safe areas, without any restrictions. The time needed to move from the centre of the playing area to either of the safe areas was approximately 1 s.

The enemy ships appeared in different horizontal lines, descending from the top of the screen down to the player’s line forming the enemy’s area. Enemies moved sideways, and when they reached either edge of the screen, they descended towards the player line. When one enemy ship reached the players area, it simply disappeared from the screen. The red enemy’s lasers fell vertically through the screen until reaching the participant’s spaceship or the bottom edge of the screen and then disappeared. When their fire hit the participant’s spaceship, 10 points were deducted from the participant’s score. When the participant destroyed an enemy unit, their score increased by 10 points. When there were less than 5 enemies in the enemy’s area, a random number of new enemies (between 1 and 12) appeared on the screen in the upper third of the playing area.

Auditory stimuli were presented via headphones to the participants. Each signal turned on for 5 s before turning off. During reinforced trials (A+, BX+) immediately after the offset of the signal the aversive outcome appeared, while during non-reinforced trials (E−, FG−) nothing happened. Signals A and X were counterbalanced as a bell and a clicker sound (50 dB). B was always a tone of 400 Hz (60 dB). E was white noise, F a tone of 50 Hz, and G a sci-fiction spaceship noise (50 dB). In the case of the compounds BX+ and FG−, each sound was merged in a single audio file and presented to the participants as a compound. All stimuli are available at https://osf.io/hwn5r/?view_only=7956499824e548f2b1f7dd324985ce67. In the game used in Herrera et al. (2022), where the predictive cues were visual, shooting sounds of the spaceships and sounds triggered by inflicting damage on the targets were presented. In contrast, during the game in the present experiments, participants only experienced the sound of the predictive signals and the non-predictive fillers; the shooting and damage related sounds were omitted to avoid confusion between external sounds related to the game’s dynamic and the predictive signals.

The aversive outcome used during the game was a mothership, whose imminent presence could be anticipated by signals A and BX described above. When the mothership appeared, the participant’s spaceship was immediately frozen, preventing any movements by the participant and hence forcing participants to anticipate the appearance of the mothership. The enemy ships disappeared from the screen during the presence of the mothership. The mothership always appeared from the left of the screen and stopped in the centre of the screen. Once placed in the centre of the screen, the mothership shot one laser for approximately 3 s impacting the entire playing area. If the participant’s spaceship was in the safe area, the point counter remained in green font and unchanged (Figure 1b); however, if the participant’s spaceship was in the playing area, the point counter turned red and decreased progressively until 300 points were deducted (Figure 1c). After this, the mothership disappeared from the screen and the enemy ships returned to the screen in the same position they were before the mothership appeared and the game continued. The participant’s score could never reach negative values. The program recorded the time spent in the playing area or in the safe area using 0.2-s windows.

##### Procedure

After reading and signing the consent form, participants performed a sampling testing of the headphones, ensuring that their headphones were working correctly. After that, they were requested to read the task instructions on a sheet of paper handed to them by the experimenter. The instructions read,

You are going to play a space-game in which you are piloting a yellow spaceship. You can control the spaceship with the left and right arrows on the keyboard to move it sideways. Pressing the space bar you can shoot a fire laser that will destroy the enemy’s battleships, if you manage to target them. Each time you destroy an enemy’s battleships, you will get points. Your goal is to get as many points as possible at the end of the game. Your points appear during the game in the top centre of the screen.

However, you must be careful! Your enemies can also shoot at you, and if they hit your spaceship, you will lose points. In both corners of the screen, there are two shields in which you can hide. Whilst you are hidden in the shields, you cannot be shot by the enemies, nor can you shoot them, so your score will not increase.

From time to time, a large enemy battleship will appear, and you can’t destroy it. When this large battleship appears, you cannot move, and you will lose lots of points. Your only chance to avoid this attack is by hiding your spaceship in the two shields at the corners. We have installed a radio in your spaceship that capture auditory signals of your enemy. These signals will help you to know when the large battleship will appear. If you can predict the appearance of the large battleship, you will have time to move to the shields and avoid losing points. However, not all auditory signals are helpful, and your task is to learn which signals can help you. Remember that you cannot get points when you are hiding, so you should optimise the time that you spend hiding by using the sensors.

Keep fighting until a message with “Thank you for your participation” appears in the screen. At this moment, please call the experimenter and tell him that you finished the game.

Remember, try to get as many points as possible.

May the force be with you!

After the participants read the instructions, the experimenter asked them if they fully understood them, and participants were given the opportunity to ask questions. Participants were then left alone in the cubicle to start the task.

During the training phase, there were eight blocks of four training trials. Each training block consisted of one presentation of each signal/s (A+, BX+, E−, FG−) in a random order. The inter-trial interval (ITI) was 12 ± 2 s. The training phase was followed by a presentation of X and A (counterbalanced across participants) in the absence of any outcomes. There were no instructions informing participants of the change between training and test phases. The experiment had a duration of approximately 15 min.

##### Design and data analysis

A within-subjects design was used (see Table 1). The target signal X was trained in compound with the signal B (BX), while the control signal A was trained alone. Signals A and BX were always reinforced. In addition to the trials with the signals A and BX, other non-reinforced signals (E and FG) were presented during the training phase and used as fillers.

**Table 1.** Experimental designs.

Experiment	Group	Training	Test	Expected results
1	Trace0	A+, BX+, E−, FG−	X? A?	X < A
2	Trace5	A—+, BX—+, E−, FG−	X? A?	X = A
3	Trace0	A+, BX+, E-, FG-	X? A?	X < A
	Trace5	A—+, BX—+, E−, FG−	X? A?	X = A

In the Training column each letter refers to a different auditory signal. “+” refers to the presence of the aversive outcome, “−” refers to the absence of outcome, “?” refers to a test in extinction, and “—” denotes the 5-s interval between offset of the cue and onset of the outcome in group Trace5. Auditory signals for A and X were counterbalanced across participants. The order of X and A presentations was counterbalanced across participants during the Test.

We measured how participants distributed their time between the safe and the playing areas. We recorded the dwell time in the safe area in three different periods during the game: (1) Pre-Signal: We averaged how much time participants spent in the safe area during the 5 s preceding each signal. This time was equivalent to the length of the signal (i.e., 5 s); (2) we recorded the dwell time in the safe area during the signal in 1-s bins for each signal. We expected that participants would spend time in the safe area in the presence of reinforced signals, but not during the presentation of the non-reinforced signals—during which they could accrue points; and (3) we recorded the dwell time in the safe area during the trace period in 1-s bins only for the targets Signal A and X (Experiments 2 and 3).

As an index of learning, we used a difference score that was calculated by subtracting the mean Pre-Signal dwell time from the dwell time in each 1-s bin of each Signal (see Herrera et al., 2022; also see Molet et al., 2006). A positive difference score reveals that participants spent more time in the safe area in the presence of the Signal or during the Trace than during the Pre-Signal period, indicating that participants anticipated the arrival of the aversive outcome. Consequently, we expected positive scores for the reinforced cues (A and BX). A difference score close to zero means that participants spent roughly the same time during the Pre-Signal and the Signal periods.^1^

In the last training trial, we analysed the dwell time using the difference score for each signal to ensure that (1) there were no differences between A and BX at the end of training. If both cues yielded similar control at the end of training, the differences observed during test cannot be accounted for by differences during acquisition, and consequently, that the expected weaker response to X during test was not a product of a learning deficit about the compound; (2) participants discriminated between the reinforced signals (A and BX) and the fillers (E and FG) at the end of training. Data across filler stimuli E− and FG− were pooled as Fs−, as there were no differences between them. We analysed the training data using ANOVAs with Signal (A, BX, and Fs) and Second (1–5 s) as factors. Herrera et al. (2022) observed that participants reached their maximum dwell time in the safe area during the last second of the signal; we therefore used the dwell time in the last second of the signal as the critical index to assess discrimination between the different signals.

During the test, we analysed the interaction between Signal (A vs. X) and Second (1–5 s). Critically, we analysed the last second to test the direction of such interaction (e.g., A > X means overshadowing, A = X no interaction, A < X means potentiation). If such comparison failed to reach standard significance tests (i.e., the comparison is not significant), we provide Bayes factor (BF_01_) tests to assess evidence for the null effect in the critical comparisons during test, otherwise we provided the BF_10_ as index for overshadowing. A prior Cauchy distribution (0.707) using the version 0.15 of JASP (JASP TEAM, 2021) was used. As a rule of thumb, we considered BFs above 3 as substantial evidence (see Jeffreys, 1961). Confidence intervals on partial-eta squares (90%) were computed using the software available in Nelson (2016). When the assumption of sphericity was violated, the Huynh–Feldt correction was applied in the corresponding conditions.

### Results

#### Training

**Figure 2.**
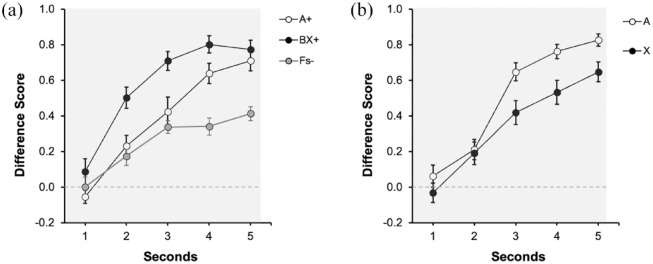
Last training and test trials of Experiment 1 with strong contiguity: (a) Training and (b) Test. Dwell time in the safe area was plotted by applying a difference score calculation, subtracting the time during Pre-Signal from the dwell time in each second of the Signal. Open circles represent the dwell time in the safe area in the presence of Signal A, the control signal, black filled circles in the presence of target Signal X, and grey circles for the non-reinforced cues. Numbers in the *x* axis represent seconds during signal. The grey rectangle symbolises the presence of the signal. Error bars represent the within-subjects standard error of the mean using O’Brien and Cousineau (2014) correction.

Figure 2a depicts the response to signals A, BX, and Fillers during the last training trial. A visual inspection of figure suggests an increase in responding to reinforced signals A and BX, peaking in the last second of the signal. Thus, participants spent more time hiding in the presence of the reinforced than the non-reinforced signals. Moreover, both reinforced signals seem to recruit similar control at the end of training. A 3 (Signal: A, BX, Fs) × 5 (1–5 s) repeated measures ANOVA revealed a significant main effect of Signal, *F*(2, 54) = 14.21, *p* < .001, η^2^_p_ = .34, 90% CI = [0.16, 0.47], Second, *F*(2.59, 69.85) = 57.08, *p* < .001, η^2^_p_ = .68, 90% CI = [0.56, 0.74] and Signal × Second interaction, *F*(6.66, 179.99) = 4.55, *p* < .001, η^2^_p_ = .14, 90% CI = [0.05, 0.20]. Subsequent analyses were focused on the last second of the signal. There were no differences when comparing both reinforced signals, *F*(1, 27) = 0.74, *p* = .396. As expected, the response to reinforced cues (pooled) was higher compared with the Fillers, *F*(1, 27) = 18.83, *p* < .001, η^2^_p_ = .41, 90% CI = [0.17, 0.57].

#### Test

Figure 2b displays the results of the test trial. Overall, A and X yielded more responding in the last second of the signal; however, responding in the presence of the target signal X was lower than in the presence of the control signal A, suggesting overshadowing with auditory signals. A 2 (Signal: A vs. X) × 5 (Second: 1–5 s) repeated measures ANOVA revealed a significant effect of Second, *F*(2.48, 67.17) = 54.32, *p* < .001, η^2^_p_ = .67, 90% CI = [0.54, 0.73], Signal *F*(1, 27) = 10.79, *p* = .003, η^2^_p_ = .29, 90% CI = [0.07, 0.47], but no interaction, *F*(45, 108) = 1.91, *p* = .114. The main effect of Signal denoted that averaged response to A (*M* = 0.66, *SD* = 0.21) was higher compared with X (*M* = 0.51, *SD* = 0.29). Despite the interaction not being significant, we analysed the effect of cue in the last second of the signal. In the last second there was an effect of Signal, *F*(1, 27) = 6.99, *p* = .013, η^2^_p_ = .21, 90% CI = [0.03, 0.40], BF_10_ = 3.59 revealing overshadowing. The Bayes factor suggests that overshadowing was 3.59 times more likely than the absence of competition.

During training, we observed that the compound BX and the control cue A yielded similar behavioural control in the last second of the signal/s. However, as Figure 2a suggests, the response to BX during the entire duration of the signal seems to be stronger compared with A, suggesting that BX recruited a faster response. This could be because cue B was intentionally of higher salience than the control/target cues A/X (counterbalanced). Although the role played by the salience of the stimuli determining the outcome of cue interactions is not entirely clear in the literature (see Urcelay, 2017), however, we followed the extended tradition to create different salience between stimuli of the compound.

Importantly, as expected, with strong temporal contiguity, cue competition was observed. This result, using auditory cues, replicates previous findings using this task with visual cues (Herrera et al., 2022). Note that this pattern of results is consistent with animal studies, in which overshadowing has been found using auditory stimuli in different preparations (e.g., Urcelay & Miller, 2009). Moreover, any potential advantage in learning for the compound BX during training disappeared when X was tested alone. In short, this experiment reveals overshadowing between auditory cues in a human predictive learning task, and hence suggests that the auditory stimuli in our protocol are suitable to detect overshadowing. Experiments 2 and 3 assessed whether weak temporal contiguity attenuates or reverts the overshadowing effect.

## Experiment 2

Experiment 1 revealed an overshadowing effect with strong temporal contiguity between the predictive auditory cues and the outcome. This result is consistent with many studies in which competition between cues has been reported, matching the predictions of associative learning models. In Experiment 2, we assessed the hypothesis that weakening of temporal contiguity between the predictive cues and the outcome attenuates such competition.

To evaluate this prediction, we used the same experimental design as in Experiment 1, except that during training a trace of 5 s was inserted between the offset of the cue/s and the onset of the outcome. Based on the results of Herrera et al. (2022), we anticipated similar behavioural control by the control cue trained alone (A) and the target cue trained in compound (X). Moreover, as noted in the introduction, if auditory stimuli were more easily configurable than visual stimuli, we may observe a shift from the absence of interactions between cues to potentiation; in that case, X should recruit a larger response than A. In our previous work, a trace of 3 s was not enough to produce potentiation, but a trace of 9 s yielded a very low rate of responding during the signal (Herrera et al., 2022). Hence, we decided to use a 5-s intermediate length of trace relative to previous experiments.

### Participants

A total of 16 undergraduate students at the University of Leicester (11 female, 5 male), with an average age of 20.62 years (*SD* = 4.91; range = 18–34) participated in the experiment in exchange for course credit. The participants had no previous experience with the task. We aimed to recruit a similar sample as in the previous experiment (*n* = 28); however, data collection was interrupted due to Covid restrictions, and we did not achieve our target sample size.

### Procedure and design

The same stimuli, task, and experimental design as described for Experiment 1 were used. The only difference relative to Experiment 1 was the temporal relationship between the predictive cues and the outcome during training. In this experiment, there was a gap of 5 s between the offset of the cue/s and the onset of the outcome. During this interval, participants could move their spaceship normally and hence gain points.

### Results

#### Training

**Figure 3.**
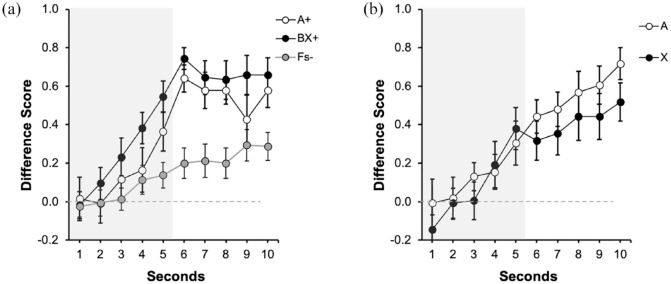
Last training and test trials of Experiment 2 with weak contiguity: (a) training and (b) test. The dwell time in the safe area was plotted by applying a difference score calculation, subtracting the time during Pre-Signal from the dwell time in each second of the Signal. Open circles represent the dwell time in the safe area in the presence of Signal A, the control signal, black filled circles in the presence of target Signal X, and grey circles for the non-reinforced cues. Numbers in the *x* axis represent seconds during signal and trace. The grey rectangle symbolises the presence of the signal. Error bars represent the within-subjects standard error of the mean using O’Brien and Cousineau’s (2014) correction.

**Figure 4.**
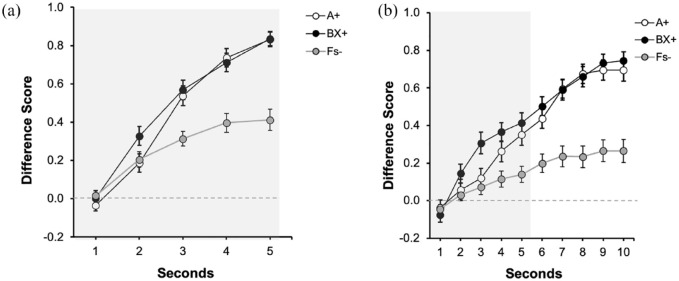
Last training trial in Experiment 3: (a) Training: Trace 0s and (b) Training: Trace 5s. Dwell time in the safe area was plotted by applying a difference score calculation, subtracting the time during Pre-Signal from the dwell time in each second of the Signal. Open circles represent the dwell time in the safe area in the presence of Signal A, the control signal, black filled circles in the presence of the compound BX, and grey circles for the non-reinforced cues (Fs). Numbers in the *x* axis represent seconds during signal and trace. The grey rectangle symbolises the presence of the signal. Error bars represent the within-subjects standard error of the mean using O’Brien and Cousineau’s (2014) correction.

**Figure 5.**
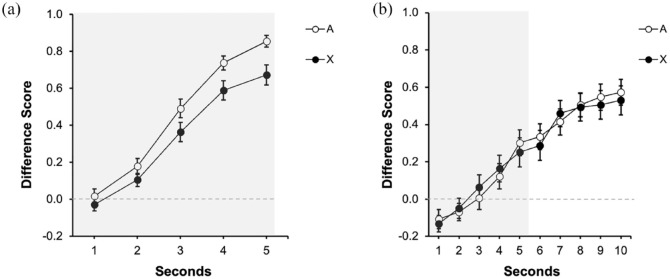
Test trial in Experiment 3: (a) Test: Trace 0s and (b) Test: Trace 5s. Dwell time in the safe area was plotted applying a difference score calculation, subtracting the time during Pre-Signal from the dwell time in each second of the Signal. Open circles represent the dwell time in the safe area in the presence of Signal A, the control signal, and filled black circles in the presence of target Signal X. Numbers in the *x* axis represent seconds during signal and trace. The grey rectangle symbolises the presence of the signal. Error bars represent the within-subjects standard error of the mean using O’Brien and Cousineau’s (2014) correction.

**Figure 6.**
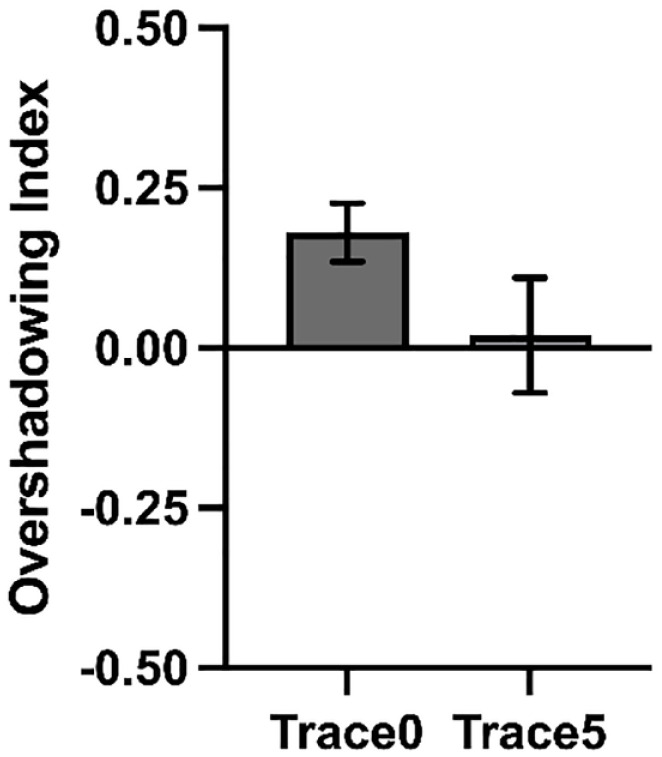
Analysis of all experiments. Each bar represents the averaged Overshadowing Index for all participants across experiments during the last second of the signal presentation. Values above 0 represent overshadowing, while a value of 0 reflects absence of overshadowing, and negative values represents potentiation. Error bars are SEM.

Figure 3a depicts the response to signals A, BX, and Fs during the last training trial. During the first 5 s, the predictive signal was present (indicated in the figure by a grey rectangle). In Figures 3, 4 and 5, 6–10 s correspond to the 5-s trace period. Overall, the behavioural control recruited by A and BX was similar, and in both cases larger compared with the Fillers. A 3 (Signal: A, BX, Fs) × 5 (Second: 1–5 s) repeated measures ANOVA carried out on the data collected during the last presentation of the signals revealed a significant main effect of Signal *F*(2, 30) = 4.25, *p* = .024, η^2^_p_ = .22, 90% CI = [0.02, 0.38] and Second, *F*(2.57, 38.56) = 9.58, *p* < .001, η^2^_p_ = .39, 90% CI = [0.16, 0.52], but no interaction between these factors, *F*(5.15, 77.23) = 1.35, *p* = .248. Importantly, in the last second of the signal, there were no differences in responding between A and BX, *F*(1, 15) = 2.91, *p* = .10, but the average rate of responding to both reinforced signals was higher than to the fillers, *F*(1, 15) = 6.10, *p* = .026, η^2^_p_ = .29, 90% CI = [0.02, 0.51].

During the trace period (6–10 s), there were no differences in responding between A and BX, *F*(1, 15) = 1.17, *p* = .296. There was a higher overall response during trace period in both reinforced signals compared with the last second of the signal, *F*(1, 15) = 6.21, *p* = .025, η^2^_p_ = .29, 90% CI = [0.02, 0.52], suggesting that the trace period recruited more behavioural control than the signal period.

#### Test

Figure 3b suggests similar performance to A and X during test, both during the signal period (1–5 s, indicated by the grey rectangle), and during the trace period (6–10 s). A 2 (Signal: A vs. X) × 5 (Second: 1–5 s) carried out on the data during the presentation of the signal revealed a significant effect of Second, *F*(1.98, 29.72) = 7.67, *p* = .002, η^2^_p_ = .34, 90% CI = [0.09, 0.49]. Neither the main effect of Signal, *F*(1,15) = 0.13, *p* = .719, nor the interaction, *F*(4, 60) = 0.68, *p* = .606, was significant. More importantly, it was not significant during the last second of the signal, *F*(1, 15) = 0.25, *p* = .627, [BF_01_ = 3.51]. The Bayes factor provided substantial support for the absence of differences between signals X and A, suggesting that the outcome of no-competition between cues was 3.51 times more likely than that of competition. If anything, the tendency was to higher response to X as anticipated by a facilitation effect. Similarly, the average responding during the trace (6–10 s) was similar across both signals, *F*(1, 15) = 2.04, *p* = .173, [BF_01_ = 1.66]. The same analysis, but constrained to the last second of the trace (in line with the analyses with the signal), revealed a similar pattern, *F*(1, 15) = 2.89, *p* = .109, [BF_01_ = 1.20]. However, it should be noted that in this case the BF only provides anecdotal evidence in favour of the null result, and this could be the result of the smaller sample used in this experiment.

The pattern of results of Experiment 2 mimicked the previous findings by Herrera et al. (2022), but using auditory cues: weakening the temporal relationship between cue and outcome attenuated overshadowing. As predicted, A and X yielded similar behavioural control during test. This pattern was observed both during the signal and trace periods, suggesting that the timing of the outcome did not influence the overlap in terms of behavioural control by both cues. Importantly, BF provided support for the absence of differences between cues, especially during the signal period. However, we did not observe a complete shift towards potentiation, as was observed using auditory stimuli in rodents (e.g., Urcelay & Miller, 2009).

Despite the results being in line with the hypothesis, the comparison between overshadowing (Experiment 1) and its absence (Experiment 2) is based on a cross-experiments comparison, making it imperative to replicate this result in a single experiment. Moreover, given that we had to stop the data collection in this experiment due to Covid restrictions, we could not recruit for Experiment 2 a sample similar to that recruited for Experiment 1. This discrepancy in terms of sample made it even more necessary to run both groups simultaneously, in a single experiment. Experiment 3 was conducted to corroborate the effect of temporal contiguity running both conditions (strong and weak contiguity) in the same experiment.

## Experiment 3

So far, overshadowing was observed with strong contiguity (Experiment 1), but weakening this temporal relationship attenuated competition, resulting in the absence of overshadowing (Experiment 2). However, this conclusion results from a cross-experiments comparison, which led us to attempt to replicate these results in a single experiment. In Experiment 3, we replicated the two previous experiments, running both groups simultaneously. Based on previous results, we anticipated the same pattern as in the two preceding experiments: overshadowing in group Trace0 (the outcome immediately followed the signal) but no overshadowing in group Trace5 (with a trace of 5 s between the offset of the signal and the onset of the outcome). Experiment 3 was preregistered: https://osf.io/hwn5r/?view_only=7956499824e548f2b1f7dd324985ce67.

## Method

### Participants

A total of 96 undergraduate students at the University of Jaén (73 female, 23 men), with an average age of 19.06 years (*SD* = 1.99; range = 18–33) participated in the experiment in exchange for course credit. The participants had no previous experience with the task. The experiment was conducted at the University of Jaén (Spain) and was approved by the Ethics committee at the University of Jaén.

We conducted a power calculation to determine the number of participants needed to detect a Group × Signal interaction during the last second of the signal. A power analysis was performed based on the effect size reported for a similar interaction (Group × Signal) in Experiment 3b by Herrera et al. (2022). The effect size described was η^2^_p_ = .084. This effect size was introduced in the software G*Power to calculate the required sample for a mixed ANOVA, with one variable between-subjects (Group) and one variable within-subjects (Signal). The software estimated a total sample of 90 participants, 45 per group (α = .05, β = .80). Given the nature of the counterbalancing, we decided to run 96 participants, 48 per group, to have an equal number of participants in all the counterbalancing subgroups.

#### Design and procedure

The design of Experiment 3 matched those in the previous two experiments: Group Trace0 was identical to the experimental group of Experiment 1, and Group Trace5 was identical to the group of Experiment 2. Data for Experiment 3 were collected at the University of Jaén (Spain); therefore, the instructions were provided in Spanish instead of English. Aside from that, all other experimental details were as described for Experiments 1 and 2.

### Results

#### Training phase

Figure 4 depicts the performance during the last training trial for both groups. The left panel (Figure 4a) displays the data of Group Trace0, corresponding to the 5 s of the presentation of the predictive cue/s. The right panel (Figure 4b) displays the data of Group Trace5 corresponding to the 5 s of the presentation of the signals (indicated by the grey rectangle, 1–5 s) and the 5 s of trace (6–10 s). Both groups showed higher response to the reinforced cues (A and BX) than to the fillers (Fs−), without differences between both reinforced cues. Overall, responding in group Trace5 (Figure 4b) was higher during trace compared with the signal period, reaching its highest level in the second immediately before the aversive outcome was presented. A 2 (Group: Trace0 vs. Trace5) × 3 (Signal: A, BX, Fs) × 5 s ANOVA carried out on the data of the last presentation of the signal (1–5 s), revealed a 3-way interaction, *F*(8, 752) = 2.69, *p* = .006, η^2^_p_ = .03, 90% CI = [0.001, 0.04]. We focused on the last second of the signal as the critical measure of learning in both groups.

As expected, in the group Trace0, there were no differences between A and BX, *F*(1, 47) = 0.01, *p* = .939, and both signals yielded higher response compared with the Fillers, *F*(1, 47) = 37.72, *p* < .001, η^2^_p_ = .45, 90% CI = [0.26, 0.57]. A similar pattern was observed in group Trace5: no differences were observed between the reinforced signals, *F*(1, 47) = 0.98, *p* = .327, and both cues collapsed recruited higher responding compared with the Fillers, *F*(1, 47) = 12.72, *p* = .001, η^2^_p_= .21, 90% CI = [0.06, 0.36].

The analysis of the trace data (6–10 s) in group Trace5 showed no differences in responding between A and BX during the overall trace interval, *F*(1, 47) = 0.19, *p* = .665. Again, we observed overall strong behavioural control during the trace compared with the last second of the signal, *F*(1, 47) = 28.03, *p* < .001, η^2^_p_ = .37, 90% CI = [0.19, 0.51].

In summary, the training data suggests that both groups learned the contingencies between signals and the outcome and there were no differences in responding between A and BX.

#### Test

Figure 5a displays participant’s performance during test in group Trace0 and Figure 5b in group Trace5. In group Trace0, there was lower response to cue X than to the control cue A, suggesting overshadowing. However, Figure 5b suggests a similar behavioural control by A and X, suggesting no overshadowing in group Trace5. The following analyses confirmed these impressions.

A 2 (Group: Trace0 vs. Trace5) x 2 (Signal: A vs. X) conducted in the last second of the signal did not reveal the expected interaction, *F*(1, 94) = 1.08, *p* = .300, although it revealed a main effect of Group *F*(1, 94) = 61.21, *p* < .001, η^2^_p_ = .39, 90% CI = [0.27, 0.49], and no effect of Signal, *F*(1, 94) = 3.43, *p* = .067. In general, responding in group Trace5 was lower compared with group Trace0. However, as Figure 5 suggests, both groups performed differently depending on which signal was tested. Although the interaction was not significant, guided by our initial pre-registered hypotheses and considering that the most informative comparison to assess overshadowing is within each group (also see the pre-registration), we analysed the last second in each group separately. In group Trace0, the effect of Signal was significant, *F*(1, 47) = 8.76, *p* = .005, η^2^_p_ = .16, 90% CI = [0.03, 0.31], [BF_10_ = 7.18] indicating overshadowing. However, as it can be appreciated in Figure 5b, group Trace5 showed a very similar response to A and X, and the effect of Signal was not significant, *F*(1, 47) = 0.21, *p* = .645 [BF_01_ = 5.76]. Moreover, the overall responding during trace was not different between signals, *F*(1, 47) = 0.03, *p* = .854 [BF_01_ = 6.27]. Similar to the signal period, an analysis restricted to the last second of the trace revealed no differences between cues, *F*(1, 47) = 0.12, *p* = .734, [BF_01_ = 6.18]. The Bayes factor provided substantial evidence for the absence of an effect either in the last second of the signal or during trace period in group Trace5.

In short, Experiment 3 replicated the findings of the previous two experiments (also see Herrera et al., 2022), showing overshadowing of auditory signals with strong temporal contiguity, but no competition after inserting a trace of 5 s.

#### Supplementary analyses

Experiment 3 revealed overshadowing in the group Trace0 and no competition in group Trace5 in the last second of the signal presentation. However, there was no significant Group by Cue interaction. Because Experiment 3 was a direct replication of Experiments 1 and 2, we decided to pool together all participants to increase the power to detect differences between groups. We calculated an Overshadowing Index for each group, subtracting the value of responding to the target cue (X) from the value of responding to the control cue (A) in the last second of the signal. A positive score is indicative of higher behavioural control by the control cue trained alone compared with the target cue trained in compound, reflecting overshadowing. An index of zero reveals no overshadowing, while negative values are indicative of potentiation.

In line with the conclusions from previous experiments, as Figure 6 shows, the Overshadowing Index was greater from zero in group Trace0, *t*(75) = 3.94, *p* < .001, *d* = 0.45, [BF_10_ = 117.93], but not in group Trace5, *t*(63) = 0.21, *p* = .830, *d* = 0.03 [BF_01_ = 7.14]. Guided by our hypothesis, an one-tailed independent *t*-test revealed that the Overshadowing Index was greater for group Trace0 compared with group Trace5, *t*(138) = 1.70, *p* = .049, *d* = 0.28 [BF_+0_ = 1.22].

## General discussion

Three experiments explored whether temporal contiguity determines the observation of cue competition in an overshadowing design using auditory cues. With strong contiguity between cues and outcome, overshadowing was consistently observed (Experiments 1 and 3): the control cue trained alone (A) yielded more behavioural control than the target cue (X) trained in compound with a competing cue (B), that is, overshadowing between auditory cues in human participants. However, weakening the cue–outcome temporal relationship during training (by inserting a 5-s trace), attenuated overshadowing, and consequently, the target and control cues (X and A, respectively) yielded similar behavioural control during the test. Notably, we observed this pattern during the presence of the signal and also during the trace intervals. The present experiments replicate the results reported by Herrera et al. (2022), but using auditory instead of visual cues as the predictive signals.

This experimental series provides further support for the notion that temporal contiguity is a determinant of cue competition (see Urcelay, 2017). The observed pattern of results is consistent with a modification of Pearce’s configural theory (Pearce, 1987) proposed by Herrera et al. (2022) in which contiguity was incorporated as a critical factor in determining whether competition is observed or not. Pearce’s theory states that animals process compound stimuli as a configural unit. When a compound, BX, is paired with an outcome, the BX configural cue acquires associative strength; subsequent tests of a fraction of the configuration, X, result in a generalisation decrement (from BX to X) and lower response to X is to be expected after configural (with BX) than elemental training (with X alone). The key element highlighted by Pearce in determining the magnitude of generalisation decrement is the similarity between the training and test cues: lower similarity can be expected between BX and X after compound training than between X and X after elemental training (in the present within-subjects design experiments, A and A, the control cue). In line with Pearce’s conception, inserting a trace between the signal and the outcome results in trace decay of the signal. However, Pearce was silent about changes in generalisation as a function of trace procedures, and Herrera et al. (2022) further assumed that in trace conditioning, the distinctive (i.e., unique) features of B and X are likely to be weaker whereas other less characteristic features (common to both elements of the compound) are likely to be better retained (e.g., a complex sound was presented but the actual frequency of the elements is difficult to recollect). Also, due to the decay, we can expect the signal to be less effective after a trace and therefore a weaker conditioned response would develop to the elements—compared with a strong contiguity (Trace0) condition where the distinctive features are likely to become predictive of the outcome. In other words, with a trace between the complex signal and the outcome, the common elements become predictive of the outcome, and this results in a broader generalisation gradient: the animal learns that a complex sound predicts the outcome, so any sound which is somewhat similar to that presented during training is likely to retrieve the outcome representation and elicit a significant level of conditioned response.

It is important to note that Pearce’s theory is a configural theory during encoding, in the sense that the organism always encodes the information in configural units but is flexible during retrieval, because responding depends on the similarity between what is trained (encoded as a configuration) and what is tested. This latter aspect of the model, in particular with the additional assumption suggested by Herrera et al. (2022), makes the model appealing to explain the current results. Configural models have traditionally been contrasted with elemental theories, such as the original Rescorla–Wagner model (Rescorla & Wagner, 1972). However, a large set of research has suggested a flexible encoding of the information, being *more* elemental or configural as a function of learning demands (see Melchers et al., 2008). In this scenario, contiguity may be another factor that modulates the degree of configural or elemental encoding. Assuming such flexibility, we propose that with strong contiguity between the compound stimulus and the outcome, the stimuli are processed in an elemental way, resulting in a large generalisation decrement and consequently in overshadowing (Experiments 1 and 3). Unlike the elemental encoding assumed to occur with strong contiguity, under conditions of weak contiguity stimuli are more likely to be encoded as a configural unit (see Urcelay & Miller, 2009). This configural processing increases the encoding of the common elements (at the expense of the distinctive features of the elements), thus attenuating generalisation decrement—that is, boosting generalisation. The increment in the similarity combined with the loss of associative strength anticipated by trace decay yielded the expected absence of competition (see Herrera et al., 2022, Table 2, for simulations). In other words, interposing a trace broadens generalisation, resulting in similar response to cues trained as part of a compound or alone.

A key aspect of our interpretation of the attenuated overshadowing observed with a trace procedure is the well-documented empirical finding that the weakening of contiguity broadens generalisation. Animal research has revealed that trace conditioning leads to poorer discrimination between CS+ and CS− than delay conditioning—where the unconditioned stimulus immediately follows or co-terminates with the conditioned stimulus (Ellison, 1964; Honey & Hall, 1992; Pavlov, 1927). We recently conducted a study in predictive learning with human participants and observed a general trend towards broader generalisation gradients with trace compared with delay conditioning (Alcalá et al., in preparation; see also Buriticá & Alcalá, 2019, for evidence in the temporal domain). Similarly, increasing the interval between sample and test in delay match to sample tasks produces a broader identification pattern (e.g., Sidman, 1969), somewhat similar to the effect of increasing the interval between training and test in other tasks (Riccio et al., 1992, 1994). In general, increasing temporal intervals results in a broader response pattern across different circumstances.

It is important to note that the similarity between BX and X is expected to increase concomitantly to the interval’s length, with longer intervals of time producing even broader generalisation. In that line, according to the rationale raised by Herrera et al. (2022), a complete shift from competition to facilitation should be observed at some point. However, our current experiments observed that both cues recruited similar behavioural control, and the Bayesian analyses supported this. Hence, in contrast with previous research with non-human animals (e.g., Urcelay & Miller, 2009), or humans (Alcalá, Kirkden, et al., 2023; Cunha et al., 2015), we did not observe potentiation between cues trained together as predictors of a delayed outcome using a trace procedure. This was slightly surprising; as we argued in the introduction, the use of auditory cues (presented through the same spatial location—the headphones) might have increased configural processing without a need to increase the trace interval. Perhaps the most parsimonious way to interpret the lack of potentiation in the present experiments is that 5 s of trace is not enough to produce this shift (but see Cunha et al., 2015; they found potentiation with a 5-s trace using a completely different task). In experiments using rats, Urcelay and Miller (2009) found potentiation with a 20-s trace, but not with 10 s, a trace at which they found neither competition nor facilitation (as in the present Experiments 2 and 3, group Trace5). However, our previous work using this task revealed that longer intervals (9 s) produced a floor effect in responding. In the present experiments, participants continued responding during the 5-s trace interval in the presence of both the control (trained by itself) and the target (trained in compound with a second element) cues. The response level during the trace was even higher than during the predictive signals themselves, probably because the participants time the appearance of the aversive outcome and aim to continue gaining points before its appearance. Hence, in this task and with the parameters used in the experiments reported here, weakening temporal contiguity attenuated competition but did not lead to facilitation.

Looking at the general picture of overshadowing and potentiation in the literature, it seems safe to conclude that different boundary conditions characterise both phenomena. While overshadowing is reasonably easy to observe across diverse experimental paradigms and species, potentiation seems to require a more selective set of parameters. However, it is worth noting that the large presence of overshadowing in the literature on compound conditioning can be simply reflecting the fact that most experiments are conducted with strong temporal contiguity between the predictive cues and the outcome, thus increasing the likelihood of cue competition. The fact that overshadowing (and its attenuation) was found with auditory stimuli in human participants parallels a large tradition of animal studies showing exactly this (e.g., Urcelay & Miller, 2009). In addition, the consistent findings using visual (see Herrera et al., 2022) and auditory stimuli (in the present experiments) suggest that the effect of contiguity goes beyond a particular set of stimuli (or a particular modality).

As far as we are aware, all the studies exploring cue interaction as a function of temporal contiguity have used elements from the same modality for the compound predictive cue: auditory in rats (Urcelay & Miller, 2009) and humans (the present experiments), flavours in rats (Batsell et al., 2012; Jensen et al., 2018), and visual in humans (Cunha et al., 2015; Herrera et al., 2022). However, there are examples in instrumental learning in which an intervening signal was found to compete with the action under strong contiguity conditions but facilitated learning about the response with weak contiguity (Alcalá, Kirkden, et al., 2023 in humans; Schatman et al., 1987 in pigeons). Assuming that the action and the intervening signal correspond to two complete separable dimensions, we might conclude that the effect of contiguity prevails even when using elements that belong to different modalities. Future research should further explore the interplay between cross-dimension compound stimuli and temporal contiguity.

Models based on reinforcement learning in which the learning mechanism is updated in a moment-to-moment basis—unlike trial-based models such as the Rescorla–Wagner model—do not include contiguity between the cue and the outcome as a factor in determining cue competition. For example, the temporal-difference model (Ludvig et al., 2012) predicts differences in overshadowing as a function of timing interval (i.e., length of cues), but only in a scenario in which the cues of the compound differ in their onset time and both cues co-terminate before the outcome (delay conditioning). Although these models do consider timing intervals as relevant for cue interactions, the temporal relationship between the predictive cues and the outcome is characterised by strong contiguity, which has a detrimental effect on learning about the target cue. Therefore, the potential differential impact of temporal contiguity on cue interactions is not directly accounted for in these models.

A few other accounts are worth mentioning here. First, based on the observation that trace-conditioned cues acquire less behavioural control with traces of a fixed duration (compared with variable traces), it has been suggested that in trace conditioning the trace is processed as another stimulus that competes with the target signal (Bonardi & Jennings, 2019). If this is the case, standard associative theories (i.e., Rescorla & Wagner, 1972) predict that in the current situation in which the target cue was trained as part of a compound, less responding should be observed, because the target cue would have two overshadowing cues (the overshadowing cue B and the trace). Alternatively, performance-based accounts (Stout & Miller, 2007) anticipate that the two competing cues (overshadowing cue and the trace) should cancel each other and more responding (i.e., potentiation) to the target should be observed. Finally, it could be possible, as anticipated by the revision of Wagner’s (1981) SOP model proposed by Dickinson and Burke (1996), that inhibitory learning took place in trace conditioning, as the CS and US would end up in different states (A2 and A1, respectively) during training, although it is unclear whether this should impact the size of the overshadowing effect as we observed in the present experiments.

The experiments reported here (see also Alcalá, Kirkden, et al., 2023; Herrera et al., 2022) suggest that temporal contiguity is a serious candidate to account for cue competition (overshadowing) and facilitation (potentiation). A growing body of evidence is now available across species, experimental protocols and learning parameters that support the key role of temporal and spatial contiguity in the outcome of compound conditioning. However, weakening of temporal contiguity not always yielded a visible attenuation of cue competition (see Alcalá, Miller, et al., 2023). This suggests that other variables, in addition to temporal contiguity, can also determine the outcome of compound conditioning experiment. Further research is needed to advance our knowledge on how concurrent signals for relevant outcomes interact.

The present pattern of results is consistent with the findings reported by Herrera et al. (2022) using visual cues in predictive and spatial tasks, suggesting that contiguity (temporal and spatial) is a key variable determining competition phenomena. In the present experiments, we did not observe a complete shift to a facilitation effect—as predicted by the modified version of the configural theory (Herrera et al., 2022; Pearce, 1987). The boundary conditions for the observation of potentiation are far less known than those for cue competition, suggesting that factors other than weak contiguity may also play a role. Future research should reveal these boundary conditions to further our understanding of competition and facilitation phenomena.

## Supplemental Material

sj-docx-1-qjp-10.1177_17470218231197170 – Supplemental material for Further evidence for the role of temporal contiguity as a determinant of overshadowing Supplemental material, sj-docx-1-qjp-10.1177_17470218231197170 for Further evidence for the role of temporal contiguity as a determinant of overshadowing by José A Alcalá, Pedro M Ogallar, José Prados and Gonzalo P Urcelay in Quarterly Journal of Experimental Psychology

## Notes

1. Complementary analyses focused on the test phase of each experiment with the raw data included in the supplementary materials. Overall, the pattern of results when analysing the raw data is similar to that reported using the difference score.
