# Supplementary material for: Further evidence for the role of temporal contiguity as a determinant of overshadowing
Source: Q J Exp Psychol (Hove). 2023 Sep 18;77(7):1375–89. doi: 10.1177/17470218231197170 (PMC11181734; doi:10.1177/17470218231197170)
Supplement: sj-docx-1-qjp-10.1177_17470218231197170 – Supplemental material for Further evidence for the role of temporal contiguity as a determinant of overshadowing [file sj-docx-1-qjp-10.1177_17470218231197170.docx]

Supplementary Material for:

Further evidence for the role of temporal contiguity as a determinant of overshadowing

José A. Alcalá^1,2^, Pedro M. Ogallar^3^, José Prados^4^, Gonzalo P. Urcelay^1^

1 University of Nottingham, UK

2 University Complutense of Madrid, Spain

3 University of Jaén, Spain

4 University of Derby, UK

Author Note

This research was supported by a UK ESRC Grant (ES/R011494/2) awarded to GPU and JP. JAA was a postdoctoral researcher associated with the Grant, and he is currently at the University Complutense of Madrid (Spain).

Corr. author: Gonzalo P. Urcelay, School of Psychology, University of Nottingham, University Park, Nottingham, NG7 2RD, UK; E-mail: [gonzalo.urcelay@nottingham.ac.uk](mailto:gonzalo.urcelay@nottingham.ac.uk)

Open Statement

De-identified data for the three experiments along with a code-book are available at DOI: 10.17639/nott.7239. Experiment 3 was pre-registered: https://osf.io/hwn5r/?view_only=7956499824e548f2b1f7dd324985ce67. All participants with valid data and observations were included in the analyses.

1. Visual representation of non-transformed data during test and most relevant comparisons.

**Experiment 1**

*Note*. *Time in seconds in the safe area in each second of the five seconds before the signal (i.e., pre period) in dashed line and during signal in solid line. Open circles represent the dwell time in the safe area in the presence of Signal A, the control signal, and filled black circles in the presence of target Signal X. Numbers in the x axis represent seconds during signal. The grey rectangle symbolizes the presence of the signal. Error bars represent the within-subjects standard error of the mean using O’Brien and Cousineau’s (2014) correction.*

There was difference between cues A and X in the last second of the signal (s5), *F*(1, 27) = 7.12, *p* = .013, η*^2^_p_* = .21, 90% CIs [.03, .40]

**Experiment 2**

*Note*. *Time in seconds in the safe area in each second of the five seconds before the signal (i.e., pre period) in dashed line and during signal in solid line. Open circles represent the dwell time in the safe area in the presence of Signal A, the control signal, and filled black circles in the presence of target Signal X. Numbers in the x axis represent seconds during signal and trace. The grey rectangle symbolizes the presence of the signal. Error bars represent the within-subjects standard error of the mean using O’Brien and Cousineau’s (2014) correction.*

There was no differences between cues A and X in the last second of the signal (s5), *F*(1, 15) = 2.14, *p* = .164, η*^2^_p_* = .12, 90% CIs [<.01, .37]

**Experiment 3**

*Group Trace0*

*Note*. *Time in seconds in the safe area in each second of the five seconds before the signal (i.e., pre period) in dashed line and during signal in solid line. Open circles represent the dwell time in the safe area in the presence of Signal A, the control signal, and filled black circles in the presence of target Signal X. Numbers in the x axis represent seconds during signal. The grey rectangle symbolizes the presence of the signal. Error bars represent the within-subjects standard error of the mean using O’Brien and Cousineau’s (2014) correction.*

There was difference between cues A and X in the last second of the signal (s5), *F*(1, 47) = 8.09, *p* = .007, η*^2^_p_* = .15, 90% CIs [.02, .30]

*Group Trace5*

*Note*. *Time in seconds in the safe area in each second of the five seconds before the signal (i.e., pre period) in dashed line and during signal in solid line. Open circles represent the dwell time in the safe area in the presence of Signal A, the control signal, and filled black circles in the presence of target Signal X. Numbers in the x axis represent seconds during signal and trace. The grey rectangle symbolizes the presence of the signal. Error bars represent the within-subjects standard error of the mean using O’Brien and Cousineau’s (2014) correction.*

There was no differences between cues A and X in the last second of the signal (s5), *F*(1, 47) = 0.02, *p* = .891, η*^2^_p_* = .0001, 90% CIs [<.0001, .03]
